# A kinematic model of stick‐insect walking

**DOI:** 10.14814/phy2.14080

**Published:** 2019-04-29

**Authors:** Tibor I. Tóth, Silvia Daun

**Affiliations:** ^1^ Department of Biology Faculty of Mathematical and Natural Sciences Heisenberg Research Group of Computational Neuroscience – Modeling Neuronal Network Function University of Cologne Koeln Germany; ^2^ Jülich Research Center Institute of Neuroscience and Medicine INM‐3 Koeln Germany

**Keywords:** Intra‐ and interleg coordination, motor control, multi‐legged locomotion, neuromechanical model

## Abstract

Animal, and insect walking (locomotion) in particular, have attracted much attention from scientists over many years up to now. The investigations included behavioral, electrophysiological experiments, as well as modeling studies. Despite the large amount of material collected, there are left many unanswered questions as to how walking and related activities are generated, maintained, and controlled. It is obvious that for them to take place, precise coordination within muscle groups of one leg and between the legs is required: intra‐ and interleg coordination. The nature, the details, and the interactions of these coordination mechanisms are not entirely clear. To help uncover them, we made use of modeling techniques, and succeeded in developing a six‐leg model of stick‐insect walking. Our main goal was to prove that the *same* model can mimic a variety of walking‐related behavioral modes, as well as the most common coordination patterns of walking just by changing the values of a few input or internal variables. As a result, the model can reproduce the basic coordination patterns of walking: tetrapod and tripod and the transition between them. It can also mimic stop and restart, change from forward‐to‐backward walking and back. Finally, it can exhibit so‐called search movements of the front legs both while walking or standing still. The mechanisms of the model that enable it to produce the aforementioned behavioral modes can hint at and prove helpful in uncovering further details of the biological mechanisms underlying walking.

## Introduction

Legged animals show a basic and characteristic motor activity: walking, by means of which they can move around in search for food and in pursuit of other vital activities. Walking is the coordinated movement of several legs, where coordination is required between the legs but also between the segments of a single leg (interleg and intraleg coordination, respectively). Because of its vital importance, walking has intensively been studied in several different species by a large number of scholars (e.g., Hughes 1952; Wendler 1966; Wilson 1966; Pearson 1972; Delcomyn 1981; Hultborn et al. 1998; Orlovsky et al. 1999; Kaliyamoorthy et al. 2005; Rossignol et al. 2006; Hooper and Büschges 2017; Bidaye et al. 2018) They found a variety of patterns of intra‐ and interleg coordination.

In this paper, we shall be concerned with insect locomotion, which is, of course, a special form of animal locomotion. Several species have been studied: stick insect (Wendler 1966; Graham 1972, 1985; Ludwar et al. 2005; Borgmann et al. 2007, 2009; Grabowska et al. 2012; Mantziaris et al. 2017); cockroach (Delcomyn 1971; Pearson 1972; Mu and Ritzmann 2008); drosophila (Wosnitza et al. 2013; Berendes et al. 2016; Szczecinski et al. 2018), to list but a few examples. The widely observed coordination patterns of walking are in insects tripod and tetrapod and the transitional patterns between them but the coordination patterns are implemented in different species using different physiological and organizational principles (e.g., Büschges and Gruhn 2007; Tuthill and Wilson 2016). This means that in some species, central commands have a larger weight in shaping locomotion than in others, while the opposite is true in some other species. This may, in turn, result in different preferences of these coordination patterns over the various insect species. For example, in cockroaches the tripod coordination pattern is the preferred one, and the role of the peripheral sensory signals during tripod is rather small. By contrast (adult) stick‐insects walk most often in tetrapod, which produces lower walking speed than tripod, and make heavy use of the actual peripheral sensory signals in shaping the walking pattern.

Beside the experimental studies, modeling was also employed to promote a better understanding of the neuromuscular mechanisms that generate and maintain walking (e.g. Altendorfer et al. 2001; Ghigliazza et al. 2003; Ekeberg et al. 2004; Ghigliazza and Holmes 2004; Kaliyamoorthy et al. 2005; Holmes et al. 2006; Daun‐Gruhn and Büschges 2011; Daun‐Gruhn and Tóth 2011; von Twickel et al. 2011, 2012; Ayali et al. 2015). Most notably, Cruse et al. (1998, 2000); Dürr et al. (2004); Schilling et al. (2013) made substantial contributions to the field of stick insect locomotion.

In our work, we have also been using modeling as a main tool to study insect locomotion but our approach is quite different to that by the aforementioned authors. In investigating the locomotor system of the stick insect, the main object of our modeling studies, we have striven to adhere to the morphological and physiological properties found in these animals. Our previous work has culminated in a kinematic model of stick‐insect walking of the three ipsilateral legs (three‐leg model) (Tóth and Daun‐Gruhn 2016). While this model was capable of showing some of the basic characteristics of the coordination patterns of walking, such as the interleg coordination of the ipsilateral legs during tripod or tetrapod and the transition between them, it has obviously remained unsatisfactory, since the contralateral legs were missing and so no coordination between the contralateral legs could be modeled. Thus, we extended our three‐leg model to a full‐fledged six‐leg model of stick insect walking. Our main goal in this study had been to demonstrate that the six‐leg model is capable of mimicking a variety of coordination patterns of walking and other walking‐related behavioral modes. They include tripod and variants of tetrapod coordination patterns and transitional patterns between them, as well as other important properties such as stop and restart of walking, backward walking, and search movements with the front legs, as well as combinations thereof (combined behavior). Moreover, all these walking‐related behavioral modes could successfully be simulated using the *same* model by changing the values of a few input or internal variables, only. The mechanisms of the model we made use of to reproduce these properties are physiologically plausible and their existence in the animals is partly supported by direct experimental evidence (e.g., in the case of backward walking (Bidaye et al. 2014)). Hence, they can be interpreted as putative biological mechanisms that might be active in the animals themselves.

## Methods

### A short survey of the existing model of three ipsilateral legs

**Figure 1:**
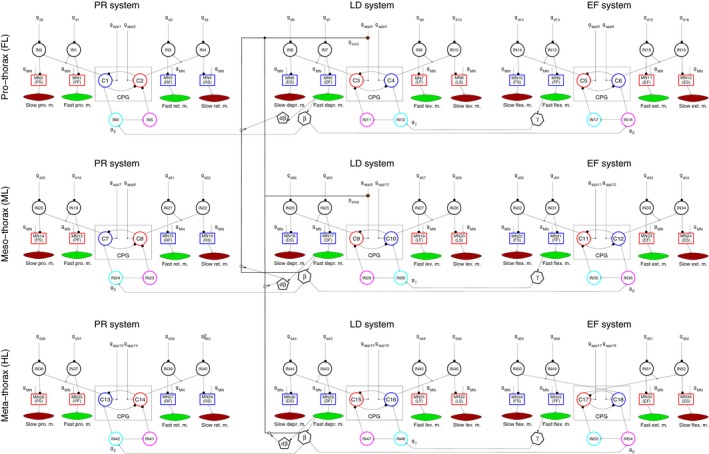
The model of walking of three ipsilateral legs (three‐leg model). Each segmental ganglion contains a connected network of three local networks. The segments are indicated on the left‐hand side of the figure: Prothorax for the front leg (FL); Mesothorax for the middle leg (ML); and Metathorax for the hind leg (HL). Each segmental network can be subdivided into three local control networks, which are associated to the main antagonistic muscle pairs of the leg: PR control network to the m. protractor‐retractor coxae (PR) pair; LD control network to the m. levator‐depressor trochanteris (LD) pair; and EF control network to the m. extensor‐flexor tibiae (EF) pair. These local control networks are, in essence, built the same way: the box CPG is the central pattern generator consisting of two mutually inhibitory nonspiking neurons (C1, C2 etc.) with centrally or peripherally driven input (excitatory synapses) with conductances *g*_app1_, *g*_app2_ etc. The boxes with MN1(PF), MN2(PS) etc. written in them symbolize the motoneurons (MNs), which activate the muscles of the corresponding muscle pair. For example, MN1(PF) is the MN of the fast protractor muscle, MN2(PS) that of the slow one. The notation follows the same rule for the other MNs, too. For example, MN18(DS) denotes the slow depressor MN of the middle leg. The MNs receive the same (central) excitatory input *g*_*M*__*N*._ The inhibitory premotor interneurons (INs) IN1, IN2 etc. convey excitation from the CPG neurons and convert it to inhibitory signals to the MNs. The conductances *g*_*d*1_, *g*_*d*2_ etc. belong to inhibitory inputs to these premotor INs. They are individually variable. Hexagons labeled by *β* or *γ*: origins of the sensory signals conveying position (*β*,* γ*), and ground contact as well as load (*β*). Pentagons labeled by *dβ*: origin of sensory signals conveying angular velocity of *β*. The conductances *g*_*β*_ and *g*_*γ*_ represent the synaptic inputs to the CPGs of the PR, LD and EF systems via the INs IN5, IN6 etc. They assume a low or a high value depending on the actual value of *β* and *γ*. The switch between the low and the high value occurs at a predefined critical value of *β* and *γ*. This is the mechanism of the intraleg coordination. Inhibitory pathways of the interleg coordination are represented by the conductances *g*_inh3_ and *g*_inh9_ at the CPG of the pro‐ and mesothoracic LD system, respectively. The small empty circles on the intersegmental pathways express the effect the angular velocity signals have on the (inhibitory) intersegmental signal flow. Arrow starting from a pentagon and pointing downwards to one of the small empty circles means that the sensory signal representing the angular velocity of *β* affects the next posterior segment (leg), an upwards pointing one that it affects the next anterior segment. Other symbols: empty triangles: excitatory, filled circles: inhibitory synapses; small filled circles: branching points of synaptic pathways; arrows from muscles to *β*,* γ* hexagons or to *dβ* pentagons indicate that the sensory signals arise because of the mechanical activity of the muscles.

Figure 1 shows a model of walking of three ipsilateral legs of the stick insect (three‐leg model). This model was constructed by the authors of the present paper (Tóth and Daun‐Gruhn 2016). The model consists of nine similar neuromuscular units. They are the control networks of the main antagonistic muscle pairs in each of the three legs attached to the muscles via motoneurons (MNs) that drive them. These main muscle pairs are: the m. protractor and m. retractor coxae, the m. levator and m. depressor trochanteris, and the m. extensor and m. flexor tibiae. The first pair of muscles is responsible for the horizontal forward‐backward movement of the leg, the second for its vertical movement, and the third pair for the flexion and extension of the leg (the movement of tibia relative to the femur). For the sake of an easier subsequent comparison with the six‐leg model, we have included the slow muscles and the corresponding slow MNs in Figure 1, even though these functional units were disabled during simulations with the three‐leg model. All local control neuronal networks have the same structure. Their core units are the central pattern generators (CPGs): small neuronal networks. The CPGs generate rhythmic (periodic) neuronal activity, which is the basis for continued walking (locomotion) in the animals. The activity of the CPGs is transmitted to the MNs via premotor interneurons (INs). The local control networks within one leg are connected by synaptic pathways and establish coordinated movement of the leg segments, which results in stepping of that leg (Tóth et al. 2012; Knops et al. 2013). Stepping of a leg is thus the result of intraleg (intra‐segmental) coordination, which enables an individual leg to carry out step movements independently of the other legs in the absence of coordination between the legs. The levator‐depressor (LD) networks of the individual legs are also connected by synaptic pathways. The activation of these connections brings about the intersegmental coordination between the ipsilateral legs.

The *intraleg* coordination works via sensory signals conveying position and force in the legs. In the model, they are represented by synaptic inputs to the protractor‐retractor (PR), LD, and extensor‐flexor (EF) local control networks. The actual values of the synaptic conductances (*g*_*β*_ and *g*_*γ*_) determine the strength of the synaptic input. In the model, *g*_*β*_ and *g*_*γ*_ can assume a “low” or a “high” value depending on the leg position, and implicitly on the force (ground contact). For example, the input to the PR system switches to its “high” value shortly before and at ground contact of the tarsus of the leg. More precisely, the switch occurs at a predefined threshold or critical value of the levation angle *β*, while *β* is decreasing toward its minimal value (ground contact).

The *interleg* coordination is based on similar principles. There, however, the direction of the leg movement (angular velocity) also plays a part in the coordination. The synaptic pathways are in this case from the meta‐thoracic to the mesothoracic ganglion and directly or indirectly to the prothoracic one. The pathways terminate on the levator CPG neurons C3 and C9. The synaptic activity (given by the conductances *g*_inh3_ and *g*_inh9_) is changed by the posterior leg's vertical position *β* and its angular velocity (vertical direction of its movement). The temporal patterns of these synaptic activities bring about the required coordination between the legs.

The three‐leg model was developed gradually, starting from a single local control network of the LD system of the middle leg (Borgmann et al. 2011; Daun‐Gruhn et al. 2011; Tóth and Daun‐Gruhn 2011). This model was extended to include the PR and EF local control networks of the middle leg (Tóth et al. 2012; Knops et al. 2013). These models could provide mechanisms underlying backward and curve walking of the stick insect. Note that because of this development strategy, we had at each stage only a few free system parameters whose values could relatively easily be determined in the simulations. Eventually, the three‐leg model could, on the ipsilateral side, produce the usual coordination patterns (tetrapod and tripod), and the transition between them (Tóth and Daun‐Gruhn 2016). Details of the properties and capabilities of this model can be found in the paper just cited, as well as in the papers Tóth and Daun (2017); Tóth et al. (2017).

The segmental LD control networks play a crucial role in the interleg coordination during walking. Timed temporal inhibition of the anterior LD central pattern generators (CPGs) brings about coordinated liftoff and touchdown of the three legs. The mechanism is somewhat different for tripod and tetrapod. It is more complex for the latter coordination pattern. This model was used in the simulations in which we studied the possible effects of decoupling one of the legs from the coordination mechanism of the three legs (Tóth and Daun 2017).

### Extending the three‐leg model to a full‐fledged six‐leg model

**Figure 2:**
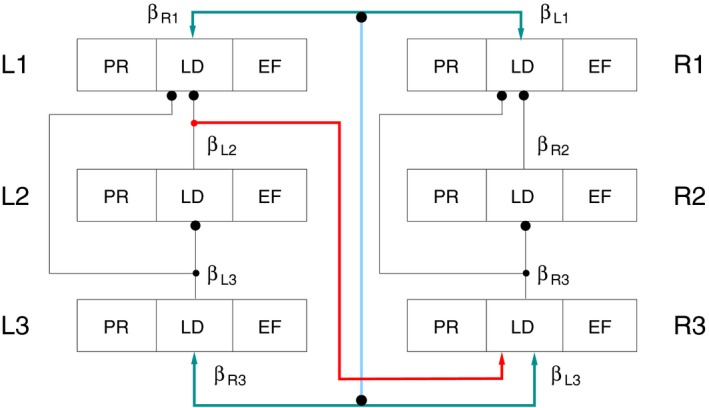
Schematic illustration of the six‐leg model and its contralateral connections. The six‐leg model is essentially a duplication of the three‐leg model. L1‐L3: left side; R1‐R3: right side. Each of them is a copy of the three‐leg model shown in Figure 1. Here, only the interleg connections are shown explicitly but somewhat simplified. The legs are represented by a triple of quadrilaterals each where the boxes labeled by PR, LD and EF stand for the PR, LD and EF systems in Figure 1. Ipsilateral black connecting lines between the legs: synaptic pathways that convey sensory signals between the legs. Their interaction brings about the interleg coordination on one side. Arrow‐heads: excitatory connections; large black filled circles: inhibitory synapses; small black filled circles: branching points of synaptic paths; *β* beside interleg pathways indicate the sensory information that pathway carries (e.g., *β*_*R*3_ near to the deep cyan pathway to L3 means that the pathway carries sensory information representing the value of *β*_*R*3_). Red line: contralateral excitatory synaptic connection from the LD system of L2 to the levator CPG neuron of R3. Deep cyan lines: bidirectional synaptic connections between the LD systems of L1 and R1, and L3 and R3, respectively. They convey excitation to the LD system of the other leg of the same segment in either direction (e.g., from L1 to R1) depending on the values of *β* and its angular velocity at the two legs. Light blue vertical line: bidirectional inhibitory synaptic connection between the pathways L1 ⟷ R1 and L3 ⟷ R3. For more explanation, see Results.

**Figure A1:**
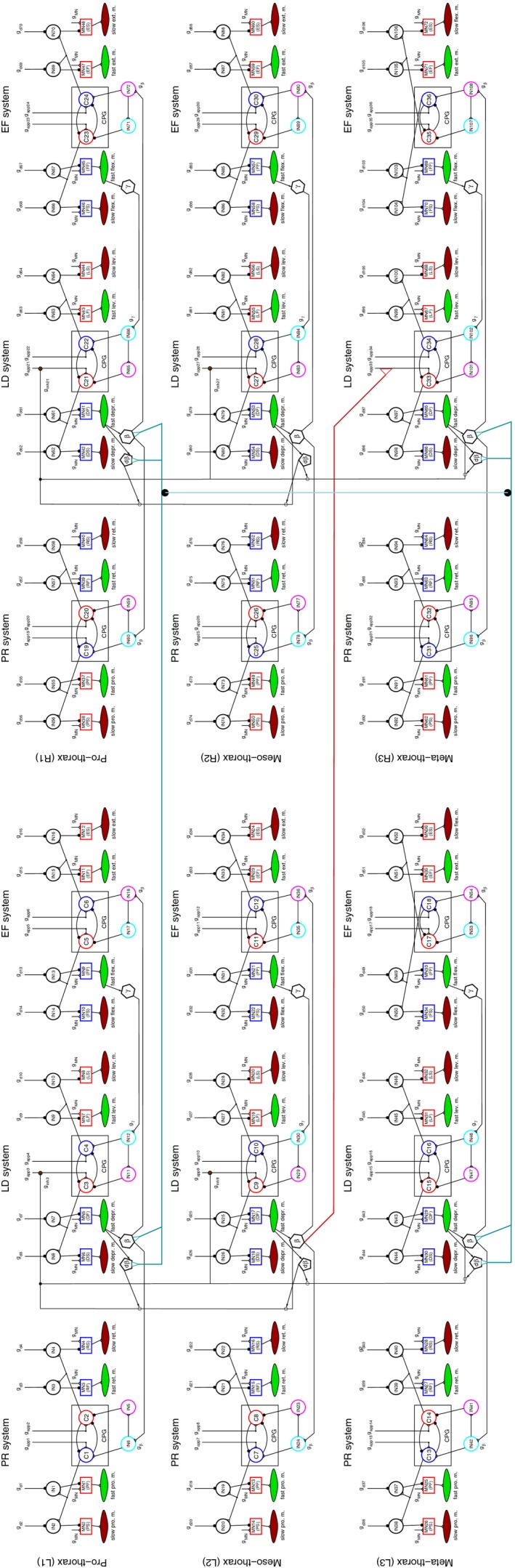
The six‐leg model in detail. Here, it is displayed how the two to three‐leg models are connected. The notations on the left‐hand side adhere to those in Figure 1. On the right‐hand side, the structure of the neuro‐muscular network is the same as on the left‐hand side but the numbering of the neurons of the different neuron types is consecutive. The red, deep cyan, and light blue lines have the same role and meaning as in Figure 2. For more explanation, see Results.

We constructed the six‐leg model by simply duplicating the three‐leg model. Its schematic illustration is shown in Figure 2. The detailed network of the full model is displayed, for the sake of completeness, in the Appendix as Fig. A1. In Figure 2, we have put emphasis on showing the interleg connections which are essential in shaping the *interleg* coordination of the legs. For the sake of simplicity, we omitted from Figure 2 the sensory signals produced by the angular velocity of the *β* angles of the individual legs. They can, however, readily be identified in Figure 1 (and Fig. A1). In addition to the duplication of the three‐leg model, we established a few connections between the contralateral sides on the basis of exploratory simulations. Detailed explanations of the function and role of these contralateral connections are provided, together with the corresponding simulation results, in the Results section.

We used the following notation to identify the legs. We denoted the left front leg by L1, the left middle leg by L2, and the left hind leg by L3. The same type of notation also applies to the right legs: replace “L” by “R”. We can now easily refer to the local control neuronal networks of the individual legs, for example, the PR system of R2 etc (Fig. 2).

The specific contralateral connections we introduced in the six‐leg model are

- an excitatory synaptic pathway from the origin of the sensory signal conveying vertical position and ground contact (*β* angle) of L2 to the levator CPG neuron of R3 (red connection in Fig. 2);
- a bidirectional synaptic connection that couples, in some sense synchronizes, the vertical position (*β*) and the corresponding angular velocity signals of L1 and R1 (upper deep cyan connection in Fig. 2);
- a similar bidirectional synaptic connection between the local control networks of L3 and R3 (lower deep cyan connection in Fig. 2);
- a bidirectional inhibitory synaptic connection between the preceding bidirectional excitatory connections (light blue line connecting the two deep cyan connections in Fig. 2).

Note that the above bidirectional connections do not contain neurons explicitly. Neurons were omitted for the sake of clarity. Another reason for omitting neurons from these pathways is that the location of such neurons within the locomotor network of the stick insect is, at present, unknown. Nevertheless, neurons belonging to the bidirectional pathways can be included without difficulty in the model by using mutually inhibitory interneurons, which allow activity of the pathway in one direction, only, at the same time.

### Implementation of the six‐leg model

The six‐leg model has 18 CPGs, 72 (slow and fast) MNs, and altogether 108 INs. The parameter values on the left‐hand side are almost identical with those on the right‐hand side. The only exception is the aforementioned excitatory pathway from L2 to R3. The parameter values were taken from earlier models, going back to the model of a single muscle pair and its control network (Borgmann et al. 2011; Daun‐Gruhn 2011; Daun‐Gruhn and Tóth 2011; Daun‐Gruhn et al. 2011; Tóth and Daun‐Gruhn 2011). Because of the stepwise extension of the model over the years, the problem of a large‐scale optimization of the parameter values in the six‐leg model did not arise.

The six‐leg model thus became a system of 648 ordinary differential equations (ODEs) of first order for the neuronal network and one second order ODE for each of the antagonistic muscle pairs (in total 18 ODEs). The model was implemented in the C language, and the ODEs were integrated by using the CVODE integration software package developed by Cohen and Hindmarsh (1996).

### Supplementary material

In order to help with the recognition and understanding of the coordination patterns and other actions during locomotion, we supply some additional material in the form of animations (videos). Note that they are solely illustrations of the simulation results, and the procedures that produced them are *not* part of the simulations with the six‐leg model. We shall refer to the corresponding specific files of the supplementary material when we present the modeling results.

## Results

### Bilateral synchronization of the stepping in the six‐leg model

**Figure 3:**
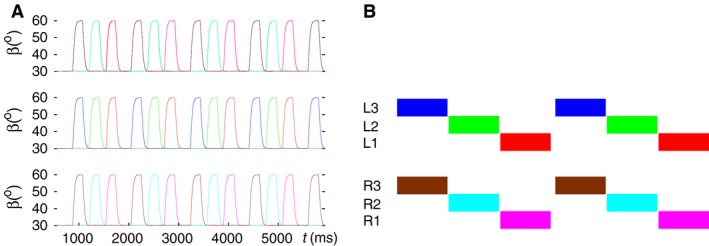
Bilateral synchronization of stepping in the six‐leg model with no contralateral connections. (A) top panel: time courses of the *β* angles of all six legs; middle panel: tetrapod of the three ipsilateral legs on the left side; bottom panel: tetrapod of the three ipsilateral legs on the right side. In all panels, *β* > 30° of a leg means swing phase of that leg. (B) schematic illustration of the two ipsilateral tetrapods. The color quadrilaterals stand for the swing phases of the legs. The color codes are in both A and B: red L1, green L2, blue L3, magenta R1, cyan R2, and brown R3. Note that every period of tetrapod walking is initiated at the hind legs on both sides. (Supplementary video: toth_daun_fig3.mp4)

First, we performed simulations using the six‐leg model with no contralateral connections and with the slow muscles and their MNs disabled (Figs. 1 and 2). The model produced bilateral synchronization of stepping in this case. This means that the leg movements on both sides were almost perfectly synchronized (Fig. 3, top panel). One can see in the middle and bottom panel of Figure 3 that each side performs a tetrapod coordination pattern of three ipsilateral legs (Fig. 3, middle panel for the left side and bottom panel for the right side). This happens because each side is in fact an autonomous three‐leg model, which is capable of producing tetrapod coordination pattern independently of the other side (Tóth and Daun‐Gruhn 2016). Since they were started at the same time and had identical initial conditions both for the neuronal and the mechanical variables, they remained synchronous indefinitely but independent of each other.

### Transforming the bilateral synchronization to tripod, tripod to tetrapod, and back to tripod coordination pattern

What we obtained in the previously described simulations is not a coordination pattern we usually see in the stick insect. Thus, the need arose to make the model produce the usual coordination patterns seen in the animal. We deemed that to achieve this would require suitable contralateral connections between the local control neuronal networks of the legs. Our strategy was to transform the bilaterally synchronous stepping into one of the usual coordination patterns. Since the ipsilateral legs on both sides performed tetrapod during bilateral synchronization, we used, on both sides, the transformation procedure to tripod as implemented in the three‐leg model (cf. Tóth and Daun‐Gruhn 2016) hoping that the required contralateral phase relations between the leg movements could also be induced by this action. It is obvious that we needed suitable contralateral connections in order to succeed. We thus tried a number of different contralateral connections with somewhat surprising outcome. At this stage of simulations with the model, we left the slow MNs and muscles in the model disabled, even though they were present, in order to have exactly the same conditions as we had in the three‐leg model (Tóth and Daun‐Gruhn 2016).

First, we connected the LD systems of L2 and R2 by an inhibitory synaptic pathway, which seemed an obvious choice; since when L2 is lifted, R2 must be on the ground and the other way around. More precisely, the lifted state of L2 evoked inhibition of the levator CPG neuron of R2.

**Figure 4:**
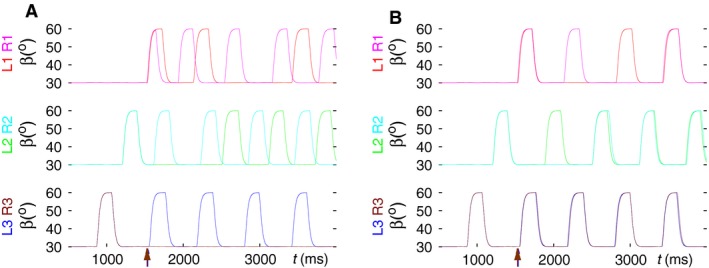
Two variants of contralateral inhibitory connections when tripod failed to emerge. (A) An inhibitory connection L2 ⟶ R2 was included in the six‐leg model. The three panels display the time courses of the *β* angles of the three pairs of contralateral legs as indicated left of each panel. The three ipsilateral legs on both sides perform tetrapod from the start on as in the three‐leg model. The transition to tripod on each side takes place as in the three‐leg model. Its start is labeled by an arrow in the bottom panel. Note that R3 remains on the ground but the front and middle legs seem, after a transitional period, to perform as if in tripod, even though R3 is not active; L1 and R2 as well as L2 and R1 lift off synchronously. (B) With the L3 ⟶ R3 inhibitory connection, the model also failed to produce tripod from the bilaterally synchronous tetrapod coordination pattern. The arrangement of the panels is the same as in A. However, the tripod failed for a different reason: the leg movements, after a transitional period, returned to the bilaterally synchronous tetrapod activity. (Supplementary videos: toth_daun_fig4A.mp4, toth_daun_fig4B.mp4)

The corresponding simulation result is displayed in Figure 4A. As it can be seen, R3 remains permanently on the ground; hence no proper coordination pattern can arise. However, the front and middle legs behave, after a transitional period, as if they were performing tripod: both L1 and R2 as well as L2 and R1 lift off and return to the ground synchronously.

Next, we established another physiologically plausible contralateral inhibitory connection from L3 to R3. As the results with this version of the six‐leg model are demonstrated in Figure 4B, the generation of a tripod coordination pattern failed in this case, too. However, the reason was different: L3 and R3 continued moving synchronously, thus the inhibition from L3 to R3 remained ineffective. This is because CPG neurons are insensitive to inhibition when they are strongly depolarized. The six legs returned eventually to the original bilaterally synchronous tetrapod coordination pattern. Making the inhibitory synaptic connection between the LD systems of L2 and R2 or L3 and R3 reciprocal did not lead to the tripod coordination pattern either (not illustrated).

We did not try the connection from L1 to R1 because they can perform search movements, apparently independently of the other four legs, while the latter legs continue walking. This makes a possible L1‐to‐R1 connection rather unlikely.

**Figure 5:**
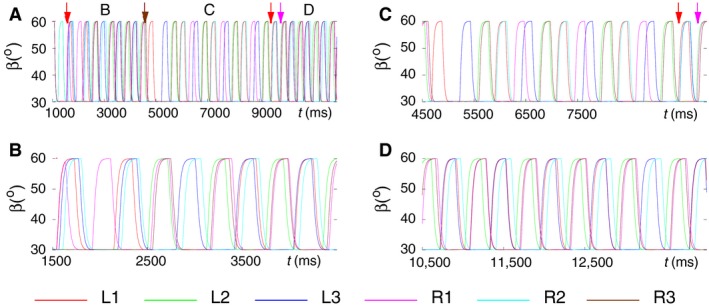
Transitions between different coordination patterns. (A) Illustration summarizing the different transitions: bilaterally synchronous walking ⟶ tripod; tripod ⟶ tetrapod, and tetrapod *→* tripod. The arrows on the top of the panel indicate the starting points of the transitions. In order to make the relation of the data in this panel to those in the subsequent panels clearer, we labeled three subintervals in this panel with B, C and D, respectively. These labels identify the position of the subsequent time intervals, which contain the same data but are displayed on a finer time scale than the one in this panel. (B) The first tripod regime. It starts almost synchronously at both hind legs at the leftmost red arrow in panel A. (C) The tetrapod regime. Its start at R3 is labeled by the brown arrow in A. The leg pairs R3‐L2, R2‐L1 and R1‐L3 are simultaneously lifted. The synchronization of the latter leg pair is not perfect, although the overlap between the swing phases is still large. The other leg pairs show an almost perfect synchronization. The transition period at the beginning of the panel is clearly visible. The arrows toward the end of the panel indicate the starting points of the transition to tripod. They are color coded to identify the leg at which the transition is initiated. In the present case, this occurs at L1 on the left side (red arrow), and R1 on the right side (magenta arrow). (D) Second tripod regime, which started at the red and magenta arrows in C. Note the fairly good synchronization of the three legs R1‐R3‐L2 and L1‐L3‐R2. The color code for the leg trajectories is shown below the panels B and D. For further details, see text. (Supplementary video: toth_daun_fig5.mp4)

We then searched for contralateral connections that involved different contralateral segments. Because of symmetry there is in fact only one possibility: connection from L2 to R3. This is, however, an excitatory one, since during tripod, both L2 and R3 are lifted at the same time. We thus established an excitatory synaptic connection that activated the levator CPG neuron of R3 when L2 was lifted. This connection enabled the model to produce a successful transition from bilaterally synchronous walking to the tripod coordination pattern. Figure 5A and B shows this result.

In Figure 5C, the successful transition to tetrapod is shown. This started ipsilaterally at R3 (brown arrow in Fig. 5A; not shown in Fig. 5C). The ipsilateral starting condition is that both the middle and hind leg move slowly, or approach the ground. That is, the angular velocity of the corresponding *β* angles is less than a small positive value. (It can be negative.) If this condition is fulfilled, the conductances *g*_app_ of the driving currents to all ipsilateral CPGs are instantaneously set to values that correspond to the tetrapod oscillatory period (and walking velocity). (See also Tóth and Daun‐Gruhn 2016).

In the six‐leg model, both sides carried out their independent transition to tetrapod according to the ipsilateral transition conditions in the three‐leg model, and a proper six‐leg tetrapod walking pattern emerged. In other words, *no* contralateral signal was necessary to bring about a tetrapod coordination pattern of good quality. During tetrapod, the leg pairs L1‐R2, L2‐R3, and L3‐R1 have synchronous swing phases. In the simulations, the overlap between the swing phases of the first two pairs of legs was practically perfect, while the overlap between the swing phases of L3 and R1 is not but it is still quite large. The emerging walking pattern can therefore be accepted as tetrapod coordination pattern. One can also see that the tetrapod coordination pattern remains stable.

The red and magenta arrows show the instants of time when the transition to tripod started at L1 and R1, respectively. The second tripod regime is displayed in Figure 5D. One can see that the synchronization between R1‐R3‐L2 and L1‐L3‐R2 is of good quality though not perfect. It is also stable, like the preceding coordination patterns, since it goes on apparently indefinitely, like the preceding ones.

The transition to tripod could only start if the ipsilateral transition conditions were fulfilled. These conditions confined the initiation of the transition to the (ipsilateral) front or hind leg, and excluded the middle leg. Furthermore, for the transition to start (at the hind or the front leg), the leg had to be lifted (*β* > *β*_min_ *)* and move upward (positive d*β/*d*t*). Once this condition was fulfilled, the conductances *g*_app_ of the driving currents (*I*_app_) to all ipsilateral CPGs were set instantaneously to their values that correspond to the tripod coordination pattern, hence walking velocity (Tóth and Daun‐Gruhn 2016). This process had to take place on both sides.

In the simulations, all combinations of contralateral front and hind legs starting the transition to tripod on their side could be produced. However, when the transition occurred at contralateral legs of different segments (e.g., at L1 and R3), no proper tripod coordination pattern emerged. Thus a kind of synchronization of the contralateral legs of the same segment was necessary. That is, if the transition started at a leg of a given segment on one side (say L1) then the transition to tripod on the contralateral side could only start when the corresponding leg (say R1) fulfilled the ipsilateral transition conditions (see above and Tóth and Daun‐Gruhn 2016). To this end, bidirectional excitatory connections between the LD systems of the contralateral front legs (L1 ⟷ R1) and of the contralateral hind legs (L3 ⟷ R3) were introduced (deep cyan lines in Fig. 2). In addition, a bidirectional inhibitory connection between the aforementioned excitatory ones was implemented in the model (light blue line in Fig. 2). These connections ensured that if one leg of a given side and given segment fulfilled the ipsilateral transition conditions first (say R3) then the transition on the contralateral side could only take place at the contralateral leg of the same segment (say L3) because of the ensuing inhibition from the activated excitatory pathway (say L3 ⟷ R3) to the pathway of the other segment (say L1 ⟷ R1), once this leg (say L3) also fulfilled the ipsilateral transition conditions on its own side. An explanation for this property of the model is that when transition to tripod is initiated at one leg (say L3) then neither of the contralateral legs (say R1, R3) are in the correct phase to produce tripod. The model therefore has to wait until the contralateral leg of the same segment (say R3) fulfills the ipsilateral transition condition and starts the transition on its own side. In this way, the correct phase difference between the two sides, needed for the tripod coordination pattern of all six legs, can be preserved. It is noteworthy that these contralateral connections and the previously mentioned L2‐R3 connection play a part at the initiation of the transition to tripod, only. During tripod, they are ineffective. Moreover, their absence during tetrapod or the transition to it does not affect this coordination pattern.

**Figure 6:**
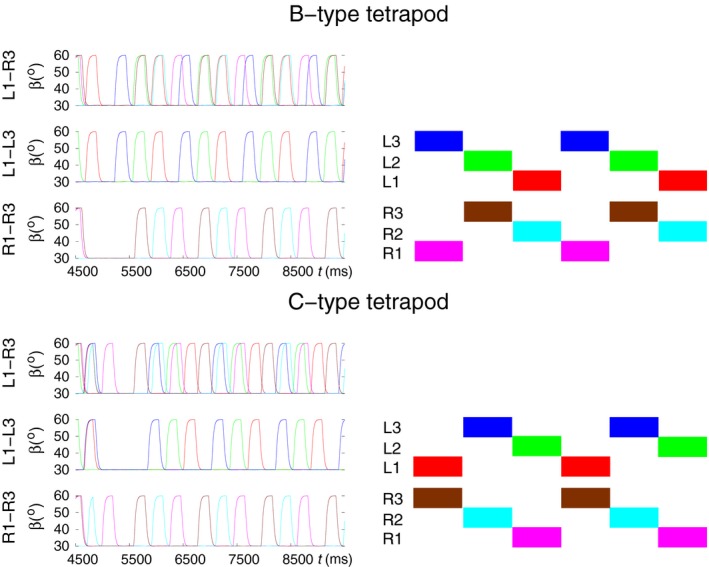
Both types of tetrapod can arise after transition from tripod to tetrapod. Left: simulation results, right: schematic illustration of the coordination patterns obtained from the experimental data by Grabowska et al. (2012). The colored quadrilaterals represent the swing phases of a given leg (e.g., L3). The two tetrapod types (B and C) are mirror images of each other with respect to the vertical plane passing through the longitudinal axis of symmetry of the animal. The color codes for the legs are the same in both schematic illustrations (right‐hand side panels) and simulation results (left‐hand‐side panels). (Supplementary videos: toth_daun_fig6B.mp4, toth_daun_fig6C.mp4)

It was observed (e.g., Grabowska et al. 2012) that a tetrapod coordination pattern and its mirror image with respect to the vertical plane passing through the longitudinal axis of symmetry of the stick insect both can occur in the same animal. In the model, we also obtained both types of tetrapod calling them B‐ and C‐type, respectively, in order to relate them to the experimental results by Grabowska et al. (2012). Figure 6 displays the simulated tetrapods of both types. The quality of the B‐type tetrapod is quite good, even the overlap between the swing phases of L3 and R1 is quite large. In comparison, the C‐type tetrapod shows a smaller overlap between the swing phases of L1 and R3.

**Figure 7:**
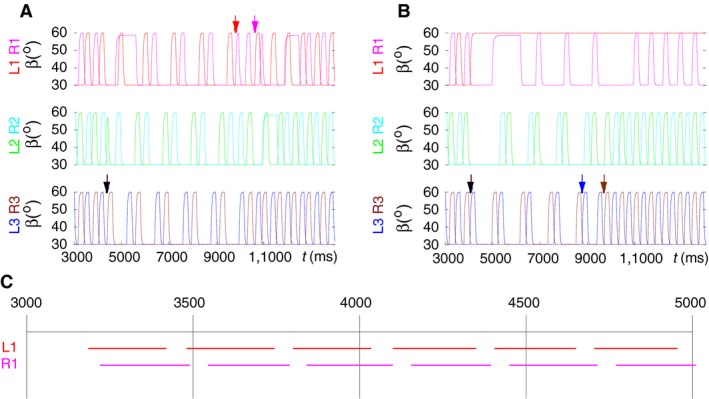
“Irregular” transitions between coordination patterns. (A) R1 remains lifted for limited periods of time after both transitions: shortly after transition to tetrapod (at ≈5000 msec) for ≈700 msec, and later after the transition to tripod (at ≈12,000 msec) for ≈500 msec; R2 remains lifted after the same transition (at ≈11,000 msec) for ≈500 msec. Note that the prolonged lifted states of L1 and R2 do not overlap in time. (B) L1 remains permanently lifted after transition to tetrapod; R1 remains lifted (at ≈5000 msec) for almost a whole period of the tetrapod walking. Note that, from the contralateral leg pairs, only the two front legs were ever simultaneously lifted. The black arrows in both A and B denote the start of the transition to tetrapod, colored arrows denote the start of the transition back to tripod. The color code corresponds to that of the legs. In A, the front legs initiate the transition, while in B the hind legs initiate the transition. (C) This panel summarizes the results of a large number of systematic simulations in which the starting point of the transition to tetrapod (from tripod) was incremented in steps of 50 msec in the time interval [3000*,* 5000] msec, which comprises more than a stepping cycle in tetrapod walking. The red lines show the intervals in which L1 behaved the regular way at and after the transition. The magenta lines show the same type of behavior for R1. The gaps between the red and magenta intervals mean irregular behavior of the corresponding leg. Note that both regular and irregular behaviors occur periodically. (Supplementary videos: toth_daun_fig7A.mp4, toth_daun_fig7B.mp4)

Up to now, we have presented simulation results that showed transitions from tripod to tetrapod and the other way around with no undesired side effects. In some cases, however, we found that one or some of the legs remained lifted for a period of time, or even permanently after transition from one coordination pattern to the other. In Figure 7, we illustrate two examples representing those “irregular” cases.

Figure 7A shows that R1 remains lifted for several hundred milliseconds after the transition to tetrapod starts. All other legs continue stepping as if in tetrapod (all panels of Fig. 7A). The second transition to tripod is initiated at the front legs (arrows in the top panel of Fig. 7A). Soon after its start, R2 and then R1 remain lifted for several hundreds of milliseconds but then return to the normal tripod stepping patterns (all panels of Fig. 7A). In the top panel of Figure 7B, one can see that L1 remains permanently lifted from the start of the transition to tetrapod. Also, R1 remains lifted for ≈1000 msec from *t *≈* *5000 msec on, that is, after the start of the transition to tetrapod. Nevertheless, the other legs seem to perform as if in a C‐type tetrapod coordination pattern. (See the *β* trajectories of the legs L2, R2 and L3, R3 in Fig. 7B.) The second transition to tripod is initiated at the hind legs (arrows in bottom panel of Fig. 7B). Again, all legs but L1 follow the stepping pattern of a tripod. Despite a partial or temporal disruption of the regular coordination patterns, these “irregular” cases do not appear completely unnatural. Stick insects also display (walking) states when one leg or two front legs remain lifted for a longer period of time (e.g., Grabowska et al. 2012). The latter is usually regarded as search movement. It is, therefore, impossible to determine whether the “irregular” cases we encountered in the simulations have natural counterparts, or are simply artifacts, undesired results.

To see how often and at what starting points such irregular behavior: L1 or R1 remains lifted for a longer period of time at the transition from tripod to tetrapod, occurs, we carried out a large number of systematic simulations in the time interval [3000*,* 5000] msec in steps of 50 msec, that is, the starting time was incremented by 50 msec at every new simulation until it reached the end point 5000 msec. From these simulation results, we constructed Figure 7C. In this panel, the red lines show the intervals in which L1 behaved the regular way (did not remain lifted), and the magenta lines where R1 did the same. The gaps between these lines are the time intervals in which the behavior of the corresponding front leg remained lifted for a longer period of time *≥* 1000 msec, or even permanently. As one can see, the regular behavior clearly dominates the irregular one, the ratio being 21:5. Moreover, both the red and the magenta intervals occur periodically, the periods being (≈236 msec) and (≈234 msec), respectively. The gaps between the colored lines are in both cases of ≈117 msec. The results of the systematic simulations show that both regular and irregular behavior occur periodically. In particular, this means that the irregular behavior is not random but systematic. Thus, random technical (numerical) artifacts can be excluded as sources for the irregular cases. Finally, we note that the irregular behavior of the middle legs (L2 and R2) occurred much more infrequently than that of the corresponding front legs, the odds being 0.33 for L2 and L1 and 0.39 for R2 and R1, respectively. Thus, the front legs clearly provided the bulk of the irregular cases.

### Enabling slow muscles and the corresponding motoneurons in the six‐leg model

**Figure 8:**
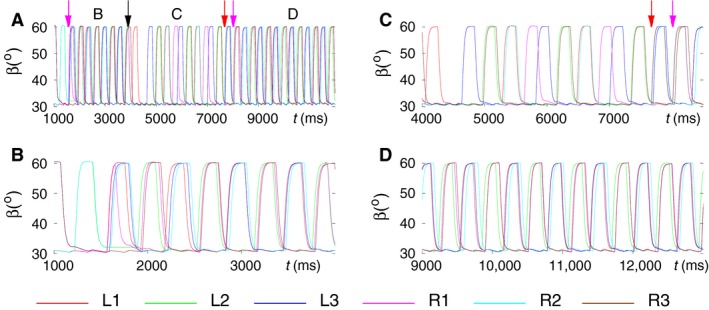
Simulation results obtained with a version of the six‐leg model in which slow MNs and muscles were enabled. The panels display the simulated tripod or tetrapod coordination patterns, expressed by the *β* trajectories of the legs, and the transitions between those patterns. The color code for the legs is displayed under the panels B and D. Thus red line: left front leg L1 etc. (A) A 13‐sec‐long simulation interval showing both coordination patterns and the transitions between them. The arrows identify the start of the transition to tripod (colored nonblack arrows) and to tetrapod (black arrow), respectively. In order to make the relation of the data in this panel to those in the subsequent panels clearer, we labeled three subintervals in this panel with B, C, and D, respectively. These labels identify the position of the subsequent time intervals, which contain the same data but are displayed on a finer time scale than the one in this panel. (B) Display of the first occurrence of tripod coordination pattern. (C) Display of the B‐type tetrapod coordination pattern, which emerges from the first tripod. (D) Return to the tripod coordination pattern. Both the tripod and the tetrapod are of good quality. The small‐amplitude oscillation near to the minimal values of the *β* time functions does not affect the quality of the coordination patterns. The color code for the leg trajectories is shown below the panels B and D. (Supplementary video: toth_daun_fig8.mp4)

As a next step in the simulations with the six‐leg model, we enabled the slow MNs and muscles in it. The properties of the slow MNs were identical to those of the fast ones, except for the “adaptation” current *I*_*q*_ whose maximal conductance *g*_*q*_ was three times as large as that of the fast MNs (cf. Daun‐Gruhn and Tóth 2011). All slow muscles had similar properties to their fast counterparts, except for the activation times (time constants), which were much longer (larger) in the slow muscles than in the fast ones. We carried out simulations with this new version of the model. Figure 8 exhibits one of the simulation results. It is clearly seen that this version of the model, too, can reproduce both coordination patterns tripod and tetrapod, as well as the transitions between them. Changing the starting times of the transitions, we also encountered here cases when L1 or R1, more rarely R2, remained temporarily, in some cases permanently lifted. These results were similar to what occurred in the simulations with disabled slow MNs and muscles (not illustrated). One can, however, notice a visible difference between the results obtained with the new version, with enabled slow MNs and muscles, and those of the previous one, with slow MNs and muscles *disabled*: in the simulation results with the new version, the base line appears to be a small‐amplitude oscillation, which is due to the activity of the slow muscles. However, this oscillation does not affect the forms of behavior that were tested with the new version of the model. In the subsequent investigations (simulations), we used this model and its extensions to mimic properties that were observed in the experiments, as described later.

### Modeling stop and restart of walking

Stop and restart of walking are important parts of the locomotion activities of insects and animals, in general. We therefore tried to reproduce these locomotion activities in the simulations with our six‐leg model. In principle, there are several possibilities to stop walking in the model. One of them is a suitable inhibition or disinhibition of the MNs via their premotor INs (cf. Fig. 1). Another basic possibility is to stop the periodic oscillatory behavior of the CPGs, which ultimately drive the MNs. When standing still, the insect must, of course, maintain a stable standing position. This is achieved by protracting and stretching the front legs and retracting and stretching the hind legs. The middle legs should also be stretched but they can point forward, sideways or backward. Obviously, all six legs must be on the ground if the highest possible stability is to be attained. Thus we endeavored to achieve these desired positions of the legs in the model, too, by both means mentioned before. We found in the simulations that the premotor INs could not produce the desired results (stop positions of the legs) in every phase of the stepping period no matter what combination of inhibition or disinhibition of the premotor INs, hence the corresponding MNs, was used. Of course, we could have tried to change the intrinsic properties of those INs and MNs to obtain the desired results but they had been used with the existing properties in several previous successful simulations (e.g., Tóth et al. 2012, 2013a, 2017; Tóth and Daun 2017). Any change in their properties could therefore have endangered the success of those previous simulations if those simulations had been repeated with the new properties of (some of) the neurons.

We therefore pursued the other possible way: directly to affect the function of the CPGs. The synaptic inputs to them were the ones where the sensory signals conveying position and vertical movement of the legs converge on the CPGs. They were the synaptic inputs *g*_*β*_ to the PR and EF systems and *g*_*γ*_ to the LD system of a leg. These synaptic pathways are the backbone of the intraleg coordination in each leg (cf. Figs. 1 and 2). We assumed that during still stand, the animal would not need a dynamic change of the synaptic activity at the input sites *g*_*β*_ and *g*_*γ*_. Thus these inputs could directly be used to impose an appropriate change of behavior on the CPGs, which in turn were to set the desired activity levels of the associated MNs. This would result in a static state of the legs. If a leg were to attain a retracted position, the corresponding retractor CPG neuron would be excited, by the synaptic current at *g*_*β*_ *,* and the CPG neuron driving the MN of the antagonistic muscle (protractor) would be inhibited by the same synaptic input (cf. Figs. 1 and 2).

If a protraction of the leg was required (in the front legs), the synaptic input *g*_*β*_ would be strongly reduced (even to zero) in order to inactivate the retractor CPG neuron. This setting of *g*_*β*_ would also ensure a disinhibition of the protractor CPG neuron, hence the corresponding MN (cf. Figs. 1 and 2). The activity levels of the LD MNs could be set by using large enough *g*_*γ*_ values. Then a robust ground contact of all legs could always be achieved. In the EF systems of the front and middle legs, a very low value of *g*_*β*_ ensured that these legs remained in the extended state. In the hind legs, however, *g*_*β*_ had to be sufficiently large in order to achieve the same result, because of the cross connection between the CPG and the premotor network.

However, the desired positions of the legs (apart from robust ground contact) could not always be produced. Thus the above method was still not sufficient to ensure that all legs attained their desired positions at the beginning of the still stand of the legs. From the simulations, it was clear that some other constraints were necessary to guarantee desired leg positions during still stand. We tried several constraints that involved the leg positions (lifted or not, angle *β*) and movement directions (angular velocities) of *β*. The simulation results, gathered in this way, suggested the condition that all right legs (R1‐R3) had to have a positive (lifting) or zero (on the ground) angular velocity in order that still stand could begin. This is the same as saying that still stand cannot begin as long as the angular velocity of *β* of any of the angles *β*_*R*1_, *β*_*R*2_, and *β*_*R*3_ violates that condition. At the first site, this seems an asymmetric constraint restricted to the right legs. In the tetrapod coordination pattern, however, there are, at any time, a pair of contralateral legs simultaneously lifted (e.g., R3‐L2, R2‐L1, and R1‐L3 in B‐type tetrapod), thus the state of a right leg (the value of the angular velocity of *β*) also reflects that of a contralateral leg. Hence, the constraint applies, in fact, to both sides. Interestingly, no constraint was necessary to initiate the restart of walking.

**Figure 9:**
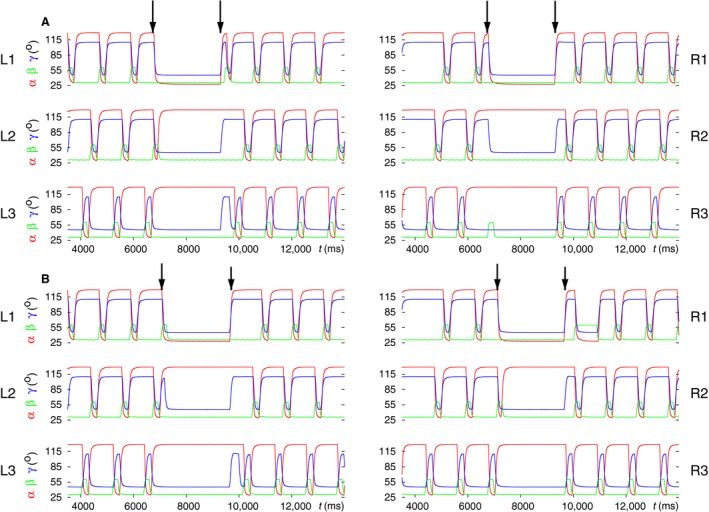
Two examples of stop and restart. In the panels, *α* (red trajectories) is the protractor‐retractor angle having its most protracted value at 28° and its most retracted one at 128°; *β* (green trajectories) is the levator‐depressor angle measuring the vertical position of the leg (tarsus) varying between 30° and 60° with 30° ≤ *β* <* *32° signaling ground contact; *γ* (blue trajectories) is the extensor‐flexor angle showing the position of the tibia relative to the femur, the most stretched position being at *γ ≈ 4*5°; the most flexed at *γ* ≈* *110°. In both cases, all legs are in the desired position: all six legs are on the ground and are stretched. In addition, L1 and R1 are protracted while the other four legs are retracted. (A) after restart, the legs walk in a C‐type tetrapod (compare the relative positions of the *β* trajectories). (B) after restart, R1 remains lifted for a period of ≈760 msec but then C‐type tetrapod emerges. Note that in both cases, the restart is initiated by lifting R3. (Supplementary videos: toth_daun_fig9A.mp4, toth_daun_fig9B.mp4)

Using the aforementioned constraint on the angular velocities of the *β* angles of R1, R2, and R3, we obtained satisfactory results in the simulations. In fact, we carried out systematic simulations over a time interval longer than a stepping period changing the beginning of the stop time in steps of 50 msec in order to test the sufficiency of the above constraint. We found that in all simulations, all legs were in the desired position. (For sake of simplicity, we chose the retracted position of the middle legs in all simulations). Samples of the results are displayed in Figure 9. In both panels, all legs assume their desired positions at the beginning of the still stand. The difference between the results in Figure 9A and B is that in the latter, one can see R1 being lifted for a prolonged period time (≈750 msec) after restart (see panel R1 of Fig. 9B). We also had cases in which L1 behaved similarly. We found that this behavior of R1 and L1 was periodic. It occurred always at the same phase of the stepping period. Hence, it is not a numerical artifact but an intrinsic property of the model. As mentioned earlier, at the transition from tripod to tetrapod or the other way around, it is, at present, impossible to determine whether this property is inherent in the stick insect. There are still two further intrinsic properties of the model that are worth mentioning.

The first one is that the restart is, without exceptions, initiated by R3 (see the green *β* trajectories indicating the swing phases of the legs in Fig. 9). The second one is that, after restart, always a C‐type tetrapod emerges never a B‐type one. Unfortunately, we could not yet solve these interesting puzzles that the model has put to us.

### Modeling search movements during walk

Grabowska et al. (2012) in their experiments noticed that the front legs of stick insects often remained lifted for a longer period of time during which these legs carried out (fast) movements. These movements, also observed by Dürr (2001); Berg et al. (2013), were termed as search movements. The main conclusion from these observations was that the front legs, while carrying out search movements, could act quite independently of the other four legs, which continued stepping as if in the tetrapod (sometimes tripod) coordination pattern.

In a further series of simulations, we employed the model to mimic search movements during walk. The main property to be demonstrated was the simultaneous display of search movements of the front legs and the continued normal walking of the other four legs. We could achieve this goal by using the premotor INs to inhibit the depressor MNs, hence inactivate the corresponding muscles and disinhibit the levator MNs (and activate the corresponding muscles) of L1 and R1 in the model, in a similar way to what was done in Tóth and Daun (2017). In addition, the same mechanism in the PR system ensured a protracted position of the front legs. Finally, we changed the driving currents (with the synaptic conductances *g*_app5_ and *g*_app6_ in L1 and *g*_app23_ and *g*_app24_ in R1) to the CPGs of the EF systems of the front legs. We changed the driving currents such as to obtain fast oscillation of the tibia of the front legs. We found in the simulations that no constraints on starting or ending the search movements were required. Thus the search movements could start and end at any instant of time. After ending the search movements, the previous B‐type tetrapod coordination pattern resumed.

**Figure 10:**
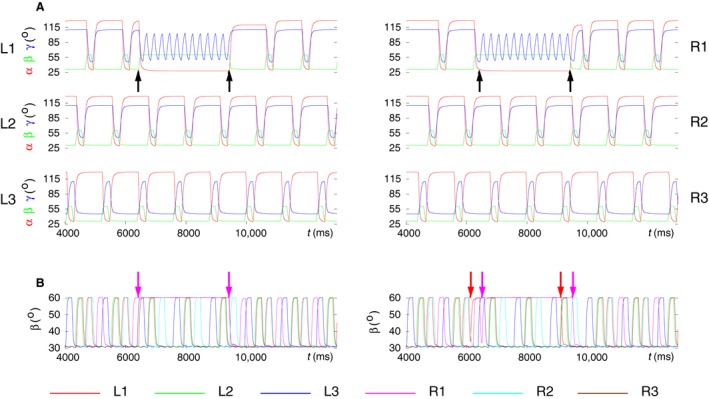
Search movements of the front legs during walking. (A) The time evolution of the angles *α*,* β,* and *γ* of each leg before, during and after search movements. The traces are identified by the labels beside the panels. Note the high‐frequency oscillation of the *γ* angles of the front legs, which causes fast movements of their tibia. The black arrows show the start and end of the search movements of the front legs. (B) The search movements of each front leg can be started independently. Here, the time courses of the *β* angles of all six legs are displayed. The left panel shows the case when the liftoff, that is, the search movements of the left and right front leg start at the same time. The traces are from the same simulation result as those in A. The other panel illustrates a case when the right front leg starts about≈500 msec later than the left front leg. The color arrows show the start and end of the search movements of either front leg. Each arrow received the same color as the *β* trace of the corresponding leg. The color code for the *β* trajectories of the legs is shown below the panels in B. (Supplementary videos: toth_daun_fig10A.mp4, toth_daun_fig10B.mp4)

Figure 10 displays typical simulation results. In Figure 10A, the time functions of the angles of the leg joints, *α*,* β* and *γ*, are displayed for each leg from L1 to R3. This result demonstrates that the search movements of front legs do not destroy the coordinated walking of the other four legs. They continue stepping as if in tetrapod irrespective of whether search movements are carried out. Figure 10A also shows the high‐frequency oscillation of the *γ* angles at the femur‐tibia joint that underlies the search movements. It is clearly seen that during search movements, both front legs are maximally protracted (*α = 2*8°) and maximally lifted (*β = 6*0°). It also demonstrates the aforementioned fact that B‐type tetrapod is restored upon ending the search movements. Figure 10B displays another example of search movements of L1 and R1 proving that search movements can be started and ended independently of each other. Also the frequency of the search movements of the individual front legs can be varied independently by appropriately choosing the driving currents to the EF CPGs of the front legs (not shown). Moreover, static position and aperiodic movements of the front legs could also be generated, although we have not yet tried the latter in the simulations.

### Modeling backward walking

Stick insects are capable of walking backward, even if they hardly use this capability. The significance of backward walking lies mainly in assisting turning when one or more legs may perform one or more steps backward to enable a fast turning (e.g., Gruhn et al. 2009). In our simulations, we did not attempt to mimic this rather complex walking behavior but restricted ourselves, for the time being, to trying to reproduce backward walking and the transition between forward‐to‐backward walking and the other way around. The main mechanism to produce backward walking was to exchange the protraction and retraction phases (time course of the *α* angles) of all legs. This was done by redirecting the active connections from the CPG to the premotor INs of the PR system (Fig. 1, Fig. A1). During forward walking, the active synaptic connections from the CPG neurons to the premotor INs of the PR system are as shown in (Fig. 1 and Fig. A1). Rosenbaum et al. (2010) showed that during backward walking the swing phase takes place during retraction, the stance phase takes place during protraction. In the model, one can achieve this easily if the original set of synaptic connections between CPG neurons and premotor INs of each PR system is inhibited (by presynaptic inhibition) and another set of synaptic connections, a kind of mirror image of the original one, from the CPG neurons to the premotor INs is activated. Thus, for example in L1, the retractor premotor INs receive synaptic excitation from the CPG neuron C1 and the protractor premotor INs from the CPG neuron C2. This kind of change takes place in every PR local control network. This mechanism is explained in more detail in (cf. Fig. 9, Tóth et al. 2012). A similar mechanism was applied to exchange the roles of the EF system of the front and hind legs during backward walking. If a transition “command” was issued in the model, then these changes in the model were effected. In addition, it proved necessary to introduce some constraints as to the effective beginning of the transition to backward walking. Thus transition to backward walking could only commence, if one of the legs L2 or R2 was lifted near to their maximal vertical position (≈60°). This is an empirical rule obtained by running a large number of simulations. Applying this condition reduced the likelihood of failed transitions to backward walking from ≈0.4 to ≈0.3 but could not eliminate it completely. At the transition from backward to forward walking, however, no constraint was necessary. The failure rate of restoring normal tetrapod forward walking was ≈0.25. The connections that were activated or inactivated during backward walking were inactivated and reactivated, respectively. (See the PR and EF local networks Fig. 1, Fig.A1, and Tóth et al. (2012)).

**Figure 11:**
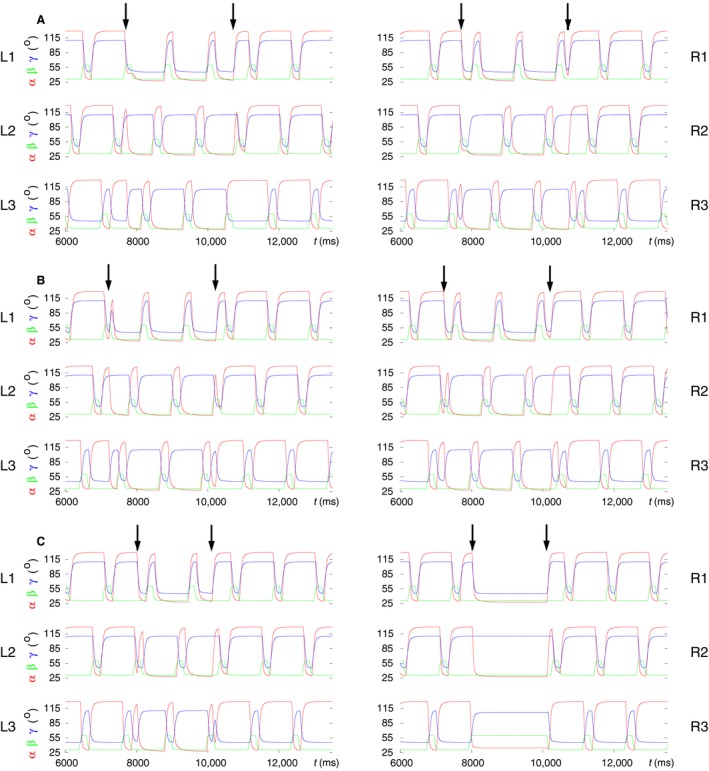
Modeling of backward walking and the transition to and from it. The time evolutions of the angles *α* (red), *β* (green) and *γ* (blue) of the leg joints of all six legs (L1‐R3) are displayed in three samples of simulation results. In all cases, the model performs B‐type tetrapod coordination pattern prior to the transition to backward walking. After returning to forward walking, three different coordination patterns arise. The intervals of backward walking are identified by pairs of arrows in the top panels of A, B, C. (A) “Common” case: B‐type tetrapod resumes upon returning to forward walking. (B) A coordination pattern of a “new type” emerges at return to forward walking. (C) Failed backward walking: the right legs R1, R2 remain on the ground, whereas R3 is lifted during the interval in which backward walking should have taken place. However, on the left side, the backward walking tetrapod pattern seems unperturbed. Note that during backward walking, the front and hind legs exchange roles. Compare the *α* and *γ* angles in those legs. In all legs, the swing and stance phases are also exchanged. However, there is no change in the time course of the *β* angle of any of the legs. (Supplementary videos: toth_daun_fig11A.mp4, toth_daun_fig11B.mp4, toth_daun_fig11C.mp4)

Figure 11 shows three samples of simulated transitions between forward and backward walking. It displays the time functions of the leg‐joint angles *α*,* β,* and *γ* of all six legs in each of the panel groups A, B, and C. The interval of backward walking is identified by a pair of arrows in each of the panels. Note, first of all, that the time course of the *β* angle of any leg is not affected by the change of walking direction. Hence, the time course of the *β* angles and the phase relations between them remain the same as in forward walking, showing basically a (B‐type) tetrapod coordination pattern.

However, the time course of the *α* angles relative to that of the *β* ones does change (see also Tóth et al. 2012). In addition, the front and hind legs exchange roles during backward walking, which involve changes in the time course of the *γ* angles of these legs. Hence, the tibiae of the front legs extend during the stance phase, and those of the hind leg flex (Fig. 11A and B) performing just the movement complementary to that during forward walking. In Figure 11C, a case is illustrated in which the transition to backward walking failed: the right legs R1, R2 remained on the ground, whereas R3 was lifted, until the end of the interval in which backward walking should have taken place, even though the left legs L1, L2, and L3 continued stepping as if during backward walking. However, the transition from this state to forward walking did succeed, even if it did not produce one of the common coordination patterns.

**Figure 12:**
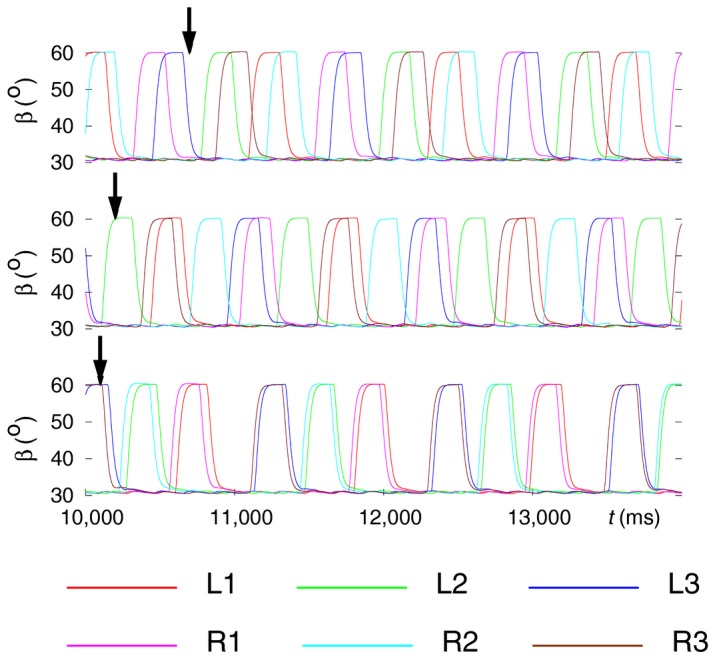
Three different coordination patterns arise in the three examples upon returning to forward walking. The panels show these coordination patterns from the same simulation examples as in Figure 11. Thus the top panel corresponds to the example in Figure 11A, the middle panel to that in Figure 11B, and the bottom panel to that in Figure 11C. By displaying the time course of the *β* angle of each of the six legs, one easily recognizes the emerging coordination patterns. Top panel: B‐type tetrapod coordination pattern. Middle panel: a “new” type of coordination patterns; here the leg pairs R3‐L1 and R1‐L3 are lifted off simultaneously, whereas R2 and L2 are lifted off alone. Bottom panel: bilaterally synchronous tetrapod (cf. Fig. 3). The arrow in each panel indicates the begin of transition from backward to forward walking. (Supplementary videos: toth_daun_fig11A.mp4, toth_daun_fig11B.mp4, toth_daun_fig11C.mp4)

The examples in Figure 11 were selected because after returning to forward walking, three different coordination patterns arose in them. From the panels of Figure 12 in which the vertical movement, the time course of the *β* angle of each of the six legs, is displayed, the coordination patterns and the difference between them can easily be recognized. The top panel of Figure 12 shows a B‐type tetrapod coordination pattern that emerges after the transition to forward walking started. The quality of the tetrapod is not quite as good as in other cases (cf., for example, Fig. 8C). Nevertheless, the tetrapod pattern is still well recognizable.

In the middle panel of Figure 12, one can see a hitherto unknown coordination pattern in which the middle legs of both sides are lifted alone, with no other leg being lifted, whereas the leg pairs R3‐L1 and R1‐L3 lift off synchronously. It is noteworthy that the simultaneous lifting of R3 and L1 appears in the C‐type tetrapod coordination pattern, whereas R1 and L3 are lifted at the same time in the B‐type tetrapod. As far as we know, no such coordination pattern in the stick insect or in other insect species, for that matter, has yet been reported neither from experiments nor produced in modeling studies.

The bottom panel of Figure 12 shows an already familiar coordination pattern: bilaterally synchronous tetrapod (cf. Fig. 3). However, in the present case, it arose in a “natural” way in the simulations as a result of the transition to forward walking, not simply because of the synchronous start of the leg movements on both sides of the symmetric six‐leg model. One should however be a bit cautious, since Figure 11C shows that all right legs (R1, R2, R3) failed to perform proper backward walking. Thus the bilaterally synchronous tetrapod occurred in rather specific circumstances, even if such circumstances are not impossible in physiological conditions.

One should also bear in mind that transition from backward to forward walking was brought about by simply restoring the synaptic connections that are active during forward walking and inactivating the ones which are used during backward walking. (See the synaptic connections between CPG neurons and the corresponding premotor INs in the PR local networks of all legs, and in the EF local networks of the front and hind legs in Figs. 1 and 2). No additional changes, inputs or synaptic drives were used, or other conditions imposed on the model. Thus the return to forward walking happened in some sense autonomously.

### Modeling combined behavior

**Figure 13:**
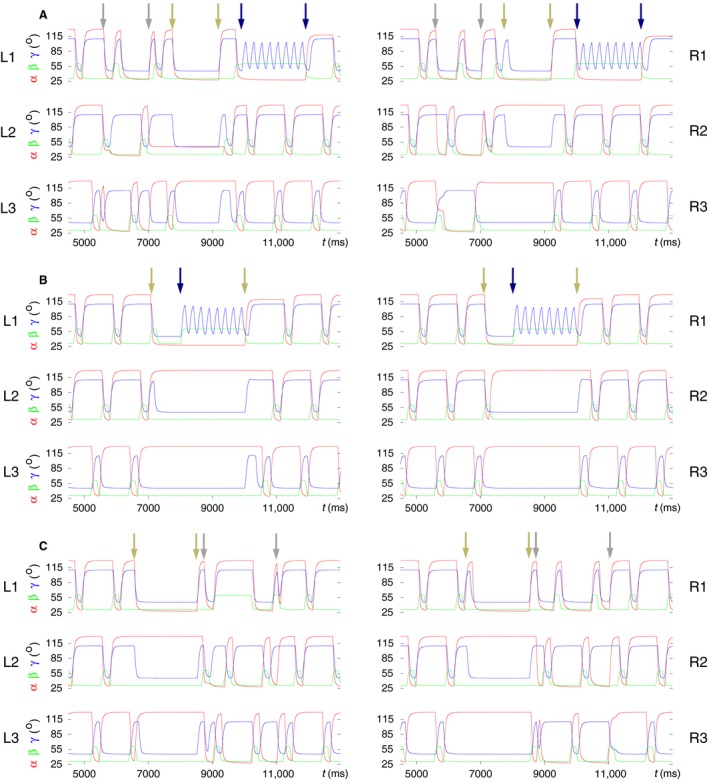
Modeling of combined behavior: a combination of behavioral modes dealt with in the preceding sections: stop and restart of walking, backward walking, search movements. The time evolutions of the angles *α* (red), *β* (green), and *γ* (blue) of the leg joints of all six legs (L1‐R3) are displayed in three samples of simulation results. In all cases, the model originally performs B‐type tetrapod coordination pattern. Then a sequence of the previously mentioned behavioral modes is evoked. The intervals where they are active are identified with arrow pairs having the same color. The color code is as follows: still‐stand interval: dark‐khaki arrows; interval of backward walking: dark‐gray arrows; interval of search movements: navy arrows. (A) A sequence of behavioral modes consisting of backward walking; followed by a still‐stand period; and the time interval of search movements (performed with the front legs). Note that during still stand, L2 is not fully retracted but rather moderately protracted (*α* ≈ 50*°*). (B) Search movements commence during the still‐stand interval, hence the two behavioral modes are partly concurrent. (C) Here, backward walking starts shortly after restart. L1 remains lifted for about 1000 msec during backward walking. Backward walking is therefore not clearly identifiable in that panel. However, in all other ones, it is clearly discernible. (Supplementary videos: toth_daun_fig13A.mp4, toth_daun_fig13B.mp4, toth_daun_fig13C.mp4)

Having succeeded in modeling specific properties of insect locomotion such as stop and restart of walking, backward walking, and search movements, we combined these properties into sequences and tested whether they still could successfully be simulated by the six‐leg model. We did not include the transition between tetrapod and tripod in this series of simulations, since we wanted first to see whether the specific properties above are compatible with the tetrapod coordination pattern, which is the most commonly used one by the stick insect. The results show that they, essentially, are. In some cases, minor changes could, however, be discerned in the still‐stand state if backward walking preceded it by a short time interval. Such a case is illustrated in Figure 13A in which L2 assumes a partially protracted position (red trajectory for *α* between the dark‐khaki arrows in the left middle panel labeled L2). In another simulation, L3 behaved the same way (not illustrated). By contrast, search movements and still stand were completely compatible, no matter in which order they appeared and whether they overlapped (Fig. 13A and B). However, backward walking and still stand could not overlap, since during still stand, the conditions for starting backward walking (*β*_*L*2_ or *β*_*R*2_ near to its maximum, 60°*)* could not be fulfilled. This is because all legs were on the ground (all *β*s between 30° and 32°) in the still‐stand condition (not illustrated).

Figure 13A displays an example in which all three specific properties (backward walking, still stand, and search movements) occurred consecutively and then terminated before the next one was evoked. One can see that apart from a minor discrepancy that L2 shows during still stand, all aforementioned specific properties could successfully be mimicked. In Figure 13B, an example is presented in which search movements start during still stand, and both behavioral modes end at the same time (third arrow from the left). The perfect compatibility between them shown here has general validity. The case in Figure 13C illustrates the behavior of the model when still stand precedes backward walking. During the latter, a slight anomaly occurred: L1 remained lifted for a period of ≈1000 msec as it can clearly be seen in panel L1 of Figure 13C (time course of *β* colored in green). Nevertheless, the other legs performed normal backward stepping. In some other cases, the so‐called “new coordination pattern” described earlier (Fig. 11B) emerged after the end of the backward walking.

**Figure 14:**
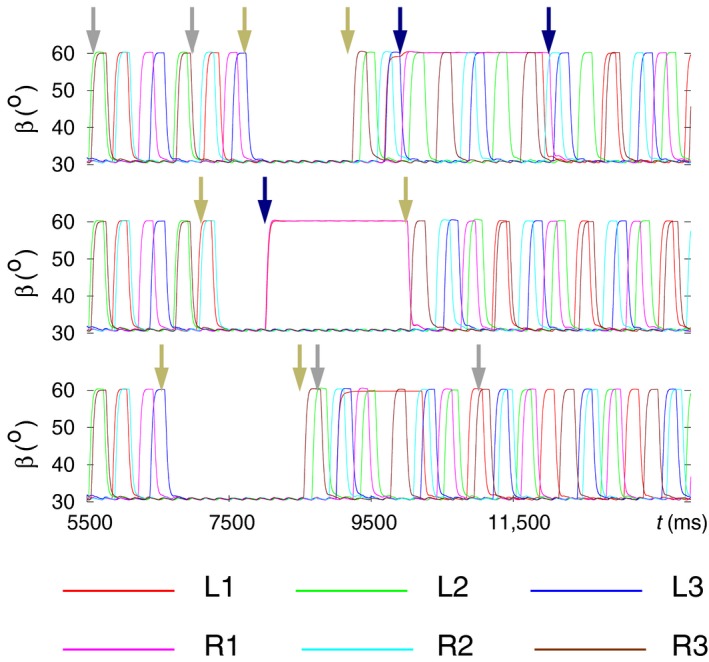
A different way of illustrating the same results as shown in Figure 13. Here, the time courses of the *β* angles of all six legs are displayed in each of the three panels. The top panel corresponds to Figure 13A, the middle one to Figure 13B, and the bottom one to Figure 13C. The arrows, having the same color codes as in Figure 13, indicate the intervals of different behavioral modes of the model. Top panel: backward walking, still‐stand period and search movements occupy disjoint intervals. Backward walking preserves the B‐type tetrapod coordination pattern (in the interval marked by the pair of dark‐gray arrows). Soon after restart, search movements are evoked, and the C‐type tetrapod is disrupted, since L1 and R1 are permanently lifted. Nevertheless, the four other legs continue stepping as if performing C‐type tetrapod (see for example, the synchronization between L3 and R2). When the search movements end, full C‐type tetrapod ensues. Middle panel: still stand occurs first and then it is combined with search movements of L1 and R1. The restart exactly coincides with the end of the period of search movements (hence, no separate second navy arrow). A clear C‐type tetrapod emerges at restart. Bottom panel: backward walking follows still stand after a short period of time. After restart, C‐type tetrapod emerges during backward walking, though the lifted leg L1 somewhat disrupts this coordination pattern. When returning to forward walking, the C‐type tetrapod improves but the synchronization between the vertical movements of the legs L1 and R3 is still not very good. (Supplementary videos: toth_daun_fig13A.mp4, toth_daun_fig13B.mp4, toth_daun_fig13C.mp4)

Figure 14 provides a different aspect of presenting the same results. Here, the time course of the *β* angle of each of the legs is displayed. This form of presentation makes the coordination patterns that appear during walking easier to recognize. However, backward walking cannot be discerned in this figure, since the *β* time courses are not affected by the change of walking direction.

In the top panel of Figure 14, three different behavioral modes appear consecutively and do not overlap. The B‐type tetrapod coordination pattern is preserved during backward walking. Upon restart, a C‐type tetrapod seems to emerge, as always after restart, but the search movements of L1 and R1 disrupt this pattern. However, the remaining four legs continue stepping as if in C‐type tetrapod. When the search movements end, the full C‐type tetrapod coordination pattern is restored. In the middle panel, still stand and search movements overlap. They are fully compatible as they do not disrupt the other concurrent behavioral mode. They end at the same time, from which a C‐type tetrapod emerges. Finally, in the bottom panel of Figure 14, the still‐stand interval is followed by that of backward walking.

In this case, L1 remains lifted for several hundred milliseconds, preventing the evolvement of the leg movements to a full‐fledged C‐type tetrapod. However, this coordination pattern eventually emerges when forward walking resumes, even though the synchronization between the legs L1 and R3 is not very good. Rarely, also B‐type tetrapod could be observed after returning to forward walking (not illustrated).

## Discussion

In the study presented here, we set out to construct a six‐leg model of locomotion of the stick insect with the aim to reproduce a variety of walking‐related behavioral modes, including, of course, coordination patterns of walking (tripod, tetrapod). To this end, we used our preexisting model of three ipsilateral legs (three‐leg model) as starting point (Fig. 1). The reproduction of these coordination patterns served as a test for the six‐leg model, since the three‐leg model could reproduce them ipsilaterally. Basically by doubling this model, to have three left and three right ipsilateral legs in the new model, we could achieve our goal (schematic illustration in Fig. 2; full network in Fig. A1). We then used this model to mimic a number of specific locomotion properties such as tripod and tetrapod coordination patterns and the transition between them, stop and restart of walking, search movements by the front legs, backward walking, and combinations thereof. We also found two additional coordination patterns that are rarely, if at all, observed in the experiments. One of them is bilaterally synchronous stepping of the contralateral legs of the same segments, that is, synchronous lifting of the legs in each of the leg pairs L1‐R1, L2‐R2, and L3‐R3. The other pattern, we simply called “new” coordination pattern in which synchronization occurs between the legs of the leg pairs L1‐R3 and L3‐R1, while L2 and R2 are lifted individually during walking (Fig. 11B). The overall quality of the simulation results has, in general, proved to be reasonably good.

### Testing various contralateral connections in the model

The six‐leg model needed only sparse contralateral connections (Fig. 2). This seems to conform to the experimental findings (e.g., Ludwar et al. 2005; Borgmann et al. 2007, 2009). Moreover, these connections were used only during transition from tetrapod to tripod, once a tetrapod coordination pattern was induced in the model (Fig. 2, Fig. A1 and Figs. 5 and 8). At this type of transition, synaptic excitation was triggered by the sensory signal representing the vertical position of L2 (*β*_*L*2_) from the LD system of L2, while L2 was lifted, to the levator CPG neuron of R3. The signal representing *β*_*L*2_ thus synchronized the vertical movements of L2 and R3 while the model entered tripod. The transition to tripod was initiated on both sides by obeying the transition conditions relating to the movement of the three ipsilateral legs in the three‐leg model. According to them, only the front or the hind leg is allowed to start the transition (Tóth and Daun‐Gruhn 2016). In addition, another type of contralateral connection (deep cyan lines in Fig. 2) ensured that the transition to tripod was started by contralateral legs of the same segment on either side. That is, if the transition happened to start first at L1 then only R1 was allowed to start the transition on the right side, provided the transition conditions of the three‐leg model were fulfilled. In no other conditions were the contralateral connections in the model used. In the initial phase of our modeling study, we tried contralateral inhibitory connections between the LD systems of L2 and R2, and L3 and R3. The connection had, of course, to be inhibitory, since, in physiological conditions, L2 and R2 are not lifted at the same time. The same is valid for the pair L3‐R3. These connections failed, since when using them, no proper coordination pattern, in particular tripod, could be generated by the model, no matter what synaptic coupling strengths were applied (Fig. 4). Thus the six‐leg model suggests that the “obvious” contralateral connections are unsuitable when modeling walking on a flat surface. This does not mean that such connections do not exist in stick insects. It merely means that they may not be active in these conditions but they may play an important part in other. However, we, at present, cannot decide based on experimental and modeling evidence whether and to what extent our assumptions on the contralateral connections might be correct.

### Modeling coordination patterns of walking and the transition between them

Turning to simulation results of specific locomotion activities, we can, first of all, state that the model succeeded in mimicking both tetrapod and tripod coordination patterns and the transition between them with sufficient accuracy (Figs. 5 and 8). The transition mechanism was simply to apply the transition conditions to tetrapod of the three‐leg model (Tóth and Daun‐Gruhn 2016) to both sides (left and right), with no additional action by, or activation of parts of the six‐leg model. In particular, no explicit contralateral entrainment of the steppings took place. A rudimentary explanation for why these two seemingly independent transitions could produce a coherent tetrapod coordination pattern could be that the original phase relations during tripod between the two sides were preserved during and after the transition to tetrapod, hence a proper tetrapod of all six legs could emerge if either side produced its own tetrapod properly.

In the simulations, the model could reproduce both types (B‐ and C‐type) of the tetrapod coordination pattern (Grabowska et al. 2012) but the B‐type appeared much more frequently than the C‐type (Fig. 6). This is in agreement with observations in the experiments. After restart from still stand, however, always C‐type tetrapod emerged. At present, we cannot explain why one or the other type of tetrapod emerged after the transition. It seems quite likely that the outcome is dependent on the phase of the stepping period at which the transition to tetrapod is initiated. However, we could not control in the simulations, for example, by choice of the starting point of transition, which type (B or C) of tetrapod coordination pattern should emerge as a result of the transition. Interestingly, we could not produce direct transitions between the two tetrapod types using the model. It seems that a transition between them should go through an intermediate tripod coordination pattern. In a more simplified model of coupled PR systems (based on Daun‐Gruhn and Tóth 2011), using phase oscillators (Yeldesbay et al. 2018), bifurcation analysis showed that the two types of tetrapod were indeed separated by the tripod coordination pattern. It appears that this property may have been preserved in the much more complex six‐leg model. There may therefore exist disjoint basins of attraction of B‐ and C‐type tetrapod here too.

In addition to tripod and tetrapod, we, on rare occasions, observed two other coordination patterns. The first was bilaterally synchronous tetrapod. The model was started in the simulations from the state in which the left and the right side performed (B‐type) tetrapod that was synchronized between the two sides (Fig. 3). However, initiating the transition to tripod this bilateral synchronization could be abolished. We also observed this coordination pattern in one case after forward walking had resumed (Figs. 11C and 12, bottom panel). Also at the same kind of transition (from backward to forward walking), we saw a “new” coordination pattern emerge (Figs. 11B and 12 middle panel). Here, also described earlier, the following pattern arose: the pair of legs L1‐R3, and separately the pair L3‐R1 were lifted synchronously, and L2 and R2 lifted off individually, while the other legs remained on the ground. We could find no reports where these coordination patterns would have been mentioned, let alone described. It can well be the case that the stick insect never uses these patterns, or cannot even produce them, but the model shows that they are theoretically possible.

### Enabling slow muscles and the corresponding motoneurons in the six‐leg model

Having succeeded in reproducing the basic coordination patterns and properties of walking and the transition between them using the fast muscles and the corresponding MNs, only, we then enabled the slow muscles and the slow MNs driving them in the model. Our main purpose of including them was to see whether the two groups of muscles and MNs are functionally compatible, that is, whether they together can reproduce the basic walking coordination patterns despite their having somewhat different properties. The expectations that they can have been born out by the successful simulation results (cf. Figs. 5 and 8). Hence, we used the model with enabled slow muscles and MNs in all subsequent simulations. In all of these simulations, they remained functionally compatible, that is, the locomotion activities could successfully be mimicked.

At the present stage, however, we did not aim at assigning specific roles of the two muscle and MN groups in the model, even though such specific roles are known from the experiments (e.g., Bässler and Stein 1996; Bässler et al. 1996). Obviously, implementing them eventually is desirable. At a later stage, this extension of the model will certainly have to be done. We think, however, that to show the compatibility of the two neuromuscular systems in the simulations for a variety of locomotion activities has been the primary requirement for the success of our present work.

### Modeling stop and restart of walking

Stop and restart of walking are important locomotion activities. They require selective activation of MNs, hence muscles. We used that fact in the simulations. We required the front legs to be protracted, the hind and middle legs to be retracted, and all legs to touch the ground and to be extended. This could be achieved using mechanisms of the intraleg coordination for this purpose. In addition, a constraint had to be imposed on the legs in order that stop could be initiated. According to this constraint, all right legs (and so all left legs that correspond to them in tetrapod) had to be on the ground or to lift off. This means nonnegative angular velocity of the corresponding *β* angles. However, no constraint was necessary at the restart of walking. (Fig. 9). As described before, the emerging tetrapod pattern upon restart was always of type C.

The simulations clearly demonstrated how a desired stationary position can be achieved in the model. They also showed that the same structural substrate (network) can be used for different functional purposes. This is not uncommon in biological systems. Of course, we do not know, at present, whether the biological “solution” follows the implementation in the model but the latter provides, at least, a hint at how this behavior might be brought about.

In an earlier work (Tóth et al. 2013b), we proposed a different mechanism of stop and restart using a single leg model. However, that mechanism was not entirely satisfactory, since the transition time from stepping to stopping the leg movement was quite long (of the order of a stepping period). The present mechanism we put forward ensures a fast stop and fast restart (≈ 200 msec), hence supersedes our old hypothesis. The present one is also more comprehensive as it involves the coordinated action of all six legs by usage of the neuronal network of the intraleg coordination mechanism in each of the legs.

### Modeling search movements

Another important locomotion property we reproduced in the simulations is the execution of search movements: keeping the front legs lifted, while they seem to explore the environment by extending and flexing their tibia. Meanwhile, the other four legs continue stepping as if in tetrapod. This is a common behavior of stick insects (Dürr et al. 2004; Grabowska et al. 2012; Berg et al. 2013).

In the model, lifting of the front legs was brought about by disinhibiting the levator MNs and simultaneously inhibiting the depressor MNs via their premotor INs (Fig. 1, Fig. A1). The fast movements of the front legs’ tibia could be achieved by changing the driving inputs to the CPGs of the EF systems of the front legs. It is not difficult to see that by appropriate choice of the driving inputs a large variety of search movements can be produced, even though, we have so far simulated periodic search movements, only. Initiating and terminating the search movements required no constraints. It is a remarkable and not a self‐evident property of our model that, during search movements of the front legs, the coordinated stepping and walking of the remaining four legs are not disrupted. Moreover, proper tetrapod is, apparently automatically, restored, most likely due to the ipsilateral interleg coordination, as soon as the search movements end. The front legs return to their regular step movements just like observed by Grabowska et al. (2012) and others in the experiments (Fig. 10). Again, very little is known about the underlying physiological mechanisms in the stick insect that shape searching behavior but substantial experimental progress has recently been made by Berg et al. (2015). Our model makes plausible suggestions, originating in modeling, on how those mechanisms might be “implemented” in the animal.

### Modeling backward walking

Stick insects hardly walk longer distances backward in natural conditions but they use their capability of doing so when turning (changing walking direction), or when navigating in complex environment (on branches of a tree). During change of walking direction, a few steps of backward walking can make turning more effective. Taking this into account, we included reproducing backward walking in our modeling tasks. During backward walking, the front and the hind legs exchange roles, the former behave like hind legs and the latter like front legs. But most importantly, the stance phase occurs during protraction and the swing phase during retraction in all legs (Rosenbaum et al. 2010). We implemented this property in our model as described in detail earlier (Tóth et al. 2012). Also the aforementioned exchange of the role of the front and hind legs during backward walking was built in the six‐leg model. With the additional constraints introduced earlier (Subsection 3.6), we succeeded in reproducing backward walking using the six‐leg model. In these simulations, we encountered two rare types of coordination patterns after returning to forward walking: a so‐called “new” type of coordination pattern, and the bilaterally synchronous tetrapod. Both of them have been described earlier in this paper (Subsection 3.6 and in the first paragraph of the Discussion). Neither of these patterns appeared in any other condition. This is a sign that the attractors of these patterns can, probably, be reached as the aftermaths of backward walking.

### Modeling combined behavior

In the final series of simulations, we combined the aforementioned types of locomotion activity in order to see whether they are compatible, that is, whether a specific activity impairs the subsequent one. The results show that search movements, and stop and restart of walking are compatible without constraints. This is an important property of the model, since it allows the search movements to start or end both during walking and during still stand. In the experiments, stick insects behaved similarly (Grabowska et al. 2012).

By contrast, the compatibility between stop and restart, and backward walking is somewhat limited. The reason for this is that the condition for starting backward walking requires L2 or R2 to be near to its maximal vertical position. This contradicts the condition that during still stand, *all* legs are on the ground. Thus restart with forward walking must first happen and only then can transition to backward walking be initiated. Also, still stand may be affected by preceding backward walking if stopping occurs almost immediately after the end of the backward walking (position of L2 in Fig. 13A). When restart precedes backward walking, a C‐type tetrapod emerges upon restart. The model, when transition to backward walking takes place, does not seem to produce good quality tetrapod in this condition. Moreover, L1 often remains on the ground for the whole length of backward walking. This is certainly a shortcoming of the six‐leg model, even though it is not a fatal one. Obviously, a better understanding of the underlying processes is required in order to remedy, in future investigations, this weakness of the present model. It is noteworthy that, after transition to forward walking, both B‐ and C‐type tetrapod can appear. These simulation results suggest that backward walking shortly before stop or after restart may affect each other in some hidden way depending on the order of their appearance.

### Possible physiological significance of the six‐leg model

In this paper, we first showed that our previous three‐leg model could successfully be extended to a six‐leg model. This new model succeeded in reproducing the *complete* tetrapod and tripod coordination patterns and the transition between them. In addition, we could simulate a number of locomotion activities that have just been mentioned and discussed in this Section. As far as we are aware of, our present six‐leg model is the first to be able to mimic all of these behavioral modes of the stick insect.

However, the question of how “physiological” our model is inevitably arises. Or put it in a different way, how much does it have common with its biological counterpart, the stick insect? To answer these questions, we first point out that the present model is the result of a chain of model developments. The chain originated from the model of a single LD system (Borgmann et al. 2011; Daun‐Gruhn et al. 2011; Tóth and Daun‐Gruhn 2011). This model of an LD system was constructed by using the latest experimental results available at that time (see References in the papers just cited). It was then gradually extended to a one‐leg model Tóth et al. (2012); Knops et al. (2013) and subsequently, to a three‐leg model (Tóth and Daun‐Gruhn 2016), the immediate predecessor of the present six‐leg model. The extensions were based on additional (and new) experimental data (e.g., Rosenbaum et al. 2010; Grabowska et al. 2012; Berg et al. 2013, 2015) and physiologically reasonable assumptions. The stepwise extensions ensured that the problem of optimizing the values of a large number (tens to hundreds) of parameters at the same time could be avoided. We basically used the same set of parameter values for all the PR, LD and EF subsystems with minor changes where they proved necessary. Most importantly, the direct relation to the biological system, the neuromuscular system of the stick insect, could be preserved throughout the development steps. We therefore think that our six‐leg model, too, can be considered to be “physiological” to the extent as our present knowledge of the stick insect's neuromuscular system allows that.

One more important point is to be noted. When we developed the model, we simply built known physiological and (synaptic) connectivity properties into it and did *not* tune it in order to simulate a specific locomotion activity (e.g., backward walking or search movements) beside the basic coordination patterns: tetrapod and tripod, as well as the transition between them. It is therefore a substantial merit of the model that it can reproduce all the specific locomotion activities presented in this paper solely by changing the properties of very few (less than 3–4 per leg out of several dozens) synaptic inputs and connections by activating or inhibiting them. This is an expression of high flexibility of the model, which is certainly a characteristic of its biological counterpart.

Emphasizing the merits of the model certainly does not mean that the correspondence between the model and the stick insect's neuromuscular system is perfect. We had to make a considerable number of assumptions and simplifications in order to succeed in modeling locomotion activities of the stick insect. These assumptions are described in detail in the Results as well as in the preceding subsections of the Discussion.

Our model of stick insect walking is certainly not universal. It cannot mimic every behavioral mode and every property the stick insect exhibits. Neither can, in fact, other models. For example, we did not investigate the dependence of the walking coordination patterns on the walking velocity (the CPGs’ oscillatory period) in the present study. We did not carry out classification of the intermediate coordination patterns, either that may occur. Inevitably, the number of problems not investigated in any given study is always much larger than that which were treated in it. But, in view of the limited time span and resources, one has always to make a choice as to which problems will be investigated. In the present study, we chose to demonstrate that a variety of walking‐related behavioral modes of the stick insect can successfully be reproduced using the *same* model. In this, we think, we have succeeded.

In the Introduction, we listed some models of the stick insect locomotion by other authors, most notably by Cruse and his coworkers (Cruse et al. 1998, 2000; Dürr et al. 2004; Schilling et al. 2013). Their approach is, however, different from ours, and simulation with it does not include some of the locomotion activities (e.g., combined behavior) we treated in this paper.

In summary, we can state that our six‐leg model makes a useful contribution to a better understanding of the stick insect's locomotion, and to six‐legged locomotion in general, even if some of the assumptions and hypotheses on which our model is based could not yet be verified by experiments. It makes a number of physiologically testable suggestions as to the organization of several aspects of locomotion (e.g., the mechanism of stop and restart of walking, and concerning the generation of search movements). It also puts forward ideas of how parts of the neuromuscular systems are used during the various locomotion activities. These principles and ideas may possibly be of relevance to other insect species, and perhaps to a far larger set of animals, despite the known shortcomings of the present model.

## Conflict of Interest

None declared.

## Supporting information




**toth_daun_fig3.mp4.** This video file illustrates the results shown in Fig. 3 of the paper; see legend of Fig. 3.

**toth_daun_fig4A.mp4.** This video file illustrates the results shown in Fig. 4A of the paper; see legend of Fig. 4A.

**toth_daun_fig4B.mp4.** This video file illustrates the results shown in Fig. 4B of the paper; see legend of Fig. 4B.

**toth_daun_fig5.mp4.** This video file illustrates the results shown in Fig. 5 of the paper; see legend of Fig. 5.

**toth_daun_fig6B.mp4.** This video file illustrates the results shown in Fig. 6, B‐type tetrapod of the paper; see legend of Fig. 6.

**toth_daun_fig6C.mp4.** This video file illustrates the results shown in Fig. 6, C‐type tetrapod of the paper; see legend of Fig. 6.

**toth_daun_fig7A.mp4.** This video file illustrates the results shown in Fig. 7A of the paper; see legend of Fig. 7A.

**toth_daun_fig7B.mp4.** This video file illustrates the results shown in Fig. 7B of the paper; see legend of Fig. 7B.

**toth_daun_fig8.mp4.** This video file illustrates the results shown in Fig. 8 of the paper; see legend of Fig. 8.

**toth_daun_fig9A.mp4.** This video file illustrates the results shown in Fig. 9A of the paper; see legend of Fig. 9A.

**toth_daun_fig9B.mp4.** This video file illustrates the results shown in Fig. 9B of the paper; see legend of Fig. 9B.

**toth_daun_fig10A.mp4.** This video file illustrates the results shown in Fig. 10A and in the left panel of Fig. 10B of the paper; see legend of Fig. 10.

**toth_daun_fig10B.mp4.** This video file illustrates the results shown in the right panel of Fig. 10B of the paper; see legend of Fig. 10.

**toth_daun_fig11A.mp4.** This video file illustrates the results shown in Fig. 11A and in the top panel of Fig. 12 of the paper; see legend of Fig. 11A and Fig. 12.

**toth_daun_fig11B.mp4.** This video file illustrates the results shown in Fig. 11B and in the middle panel of Fig. 12 of the paper; see legend of Fig. 11B and Fig. 12.

**toth_daun_fig11C.mp4.** This video file illustrates the results shown in Fig. 11C and in the bottom panel of Fig. 12 of the paper; see legend of Fig. 11C and Fig. 12.

**toth_daun_fig13A.mp4.** This video file illustrates the results shown in Fig. 13A and in the top panel of Fig. 14 of the paper; see legend of Fig. 13A and Fig. 14.

**toth_daun_fig13B.mp4.** This video file illustrates the results shown in Fig. 13B and in the middle panel of Fig. 14 of the paper; see legend of Fig. 13B and Fig. 14.

**toth_daun_fig13C.mp4.** This video file illustrates the results shown in Fig. 13C and in the bottom panel of Fig. 14 of the paper; see legend of Fig. 13C and Fig. 14.
